# Comparative lipid profiling of murine and human atherosclerotic plaques using high-resolution MALDI MSI

**DOI:** 10.1007/s00424-021-02643-x

**Published:** 2021-11-19

**Authors:** Pegah Khamehgir-Silz, Stefanie Gerbig, Nadine Volk, Sabine Schulz, Bernhard Spengler, Markus Hecker, Andreas H. Wagner

**Affiliations:** 1 Institute of Inorganic and Analytical Chemistry, Justus Liebig University, Giessen, Germany; 2 Department of Cardiovascular Physiology, Heidelberg University, Im Neuenheimer Feld 326, 69120 Heidelberg, Germany; 3 Institute of Pathology, Heidelberg University Hospital, Heidelberg, Germany; 4 Tissue Bank of the National Center for Tumor Diseases, Heidelberg, Germany

**Keywords:** MALDI imaging, Atherosclerosis, Lipid profiling, Human, ApoE

## Abstract

The distribution of atherosclerotic lesions in the aorta and its branches of ApoE knockout (ApoE^−/−^) mice is like that of patients with atherosclerosis. By using high-resolution MALDI mass spectrometry imaging (MSI), we aimed at characterizing universally applicable physiological biomarkers by comparing the murine lipid marker profile with that of human atherosclerotic arteries. Therefore, the aorta or carotid artery of male ApoE^−/−^ mice at different ages, human arteries with documented atherosclerotic changes originated from amputated limbs, and corresponding controls were analysed. Obtained data were subjected to multivariate statistical analysis to identify potential biomarkers. Thirty-one m/z values corresponding to individual lipid species of cholesterol esters, lysophosphatidylcholines, lysophosphatidylethanolamines, and cholesterol derivatives were found to be specific in aortic atherosclerotic plaques of old ApoE^−/−^ mice. The lipid composition at related vessel positions of young ApoE^−/−^ mice was more comparable with wild-type mice. Twenty-six m/z values of the murine lipid markers were found in human atherosclerotic peripheral arteries but also control vessels and showed a more patient-dependent diverse distribution. Extensive data analysis without marker preselection based on mouse data revealed lysophosphatidylcholine and glucosylated cholesterol species, the latter not being detected in the murine atherosclerotic tissue, as specific potential novel human atherosclerotic vessel markers. Despite the heterogeneous lipid profile of atherosclerotic peripheral arteries derived from human patients, we identified lipids specifically colocalized to atherosclerotic human tissue and plaques in ApoE^−/−^ mice. These data highlight species-dependent differences in lipid profiles between peripheral artery disease and aortic atherosclerosis.

**Supplementary Information:**

The online version contains supplementary material available at 10.1007/s00424-021-02643-x.

## Introduction

Cardiovascular diseases (CVDs) are still the leading cause of all deaths worldwide [55]. Atherosclerosis is the major cause of CVD and is regarded as a chronic inflammatory disease of middle-sized and large conduit arteries [46]. Because of local hemodynamic alterations, particularly coronary arterial bifurcations are susceptible to atherosclerosis [38]. Atherosclerotic plaque development and rupture may be influenced by the lipid composition within plaques, resulting in thrombotic occlusions, which account for most life-threatening acute coronary syndromes [18]. During the process of plaque progression, the plaque contents may be regarded as surrogate markers of plaque status and risk of complications [33]. Lipids constitute a heterogeneous molecular class which is classified into eight categories by the Lipid MAPS consortium [12]. The in situ investigation of this molecular class by matrix-assisted laser desorption/ionization mass spectrometry imaging (MALDI MSI), first introduced in 1994 [50], enables identification and visualization of hundreds of lipid species with a label-free and non-targeted method directly from the tissue. Since then, instrumental advancements [25, 26] have made MALDI MSI a powerful tool for the investigation and visualization of lipids [14, 22], peptides [20], biomolecules and their metabolites, pharmaceuticals, and other xenobiotics [47] in biological samples.

In the context of atherosclerosis, recent studies use MSI as a novel strategy to visualize atherosclerotic plaque localization in the aorta of atherosclerotic mice such as LDL receptor knockout (LDLR^–/–^) fed a cholesterol-rich diet or apolipoprotein E knockout (ApoE^–/–^) mice [7, 31], and to evaluate treatment effects on plaque formation and remodelling of the complex lipid components [7]. These animal studies define several lipid markers for a deeper understanding of the causes and underlying mechanisms of plaque progression. Other recent studies analyse data from a few human samples, e.g., to illustrate the potential for imaging lipid biochemistry in human atherosclerosis [53]. Recently, a 2-dimensional (2D) and three-dimensional (3D) MALDI-MSI study identified plaque-specific lipids in ApoE^−/−^ and LDLR^−/−^ mice [6]. In our study, we used MALDI MSI as a label-free and non-targeted method [45] for the direct comparison of murine and human atherosclerotic plaques to determine lipid markers for the differentiation of ApoE^−/−^ from wild-type mice and for human atherosclerotic tissue samples with differences in progression and medication which, to the best of our knowledge, has not yet been performed.

## Material and methods

### Materials

Acetone (Uvasol®), trifluoroacetic acid (Uvasol®), and 2,5-dihydroxybenzoic acid (DHB) were purchased from Merck KGaA (Darmstadt, Germany). Water (HPLC grade) was purchased from Sigma (Steinheim, Germany). Gelatine was purchased from VWR Chemicals (Leuven, Belgium).

### Murine tissue samples

Inbred male ApoE^−/−^ mice (B6.129P2-ApoE/tm1/Unc/J; stock No. 002052, The Jackson Laboratory, USA) and male wild-type (WT) control littermates were housed in the Interfaculty Biomedical Research Facility (IBF) of Heidelberg University under standard conditions with 12-h light and 12-h dark cycle. Water and chow were offered ad libitum. All animal experiments were performed with the permission of the local animal welfare committee (Regional Council Karlsruhe, Germany, permission number G-229/19) and conformed to the guidelines from Directive 2010/63/EU of the European Parliament on the protection of animals used for scientific purposes or the current NIH guidelines. Young and old ApoE^−/−^ mice (age, 14–15 weeks, *n* = 2, or 49–60 weeks, *n* = 4) and WT mice (age, 15 weeks, *n* = 2; 48–49 weeks *n* = 4) were used in this study (Supplemental Table S1). After anesthetization, the mice were euthanized by exposure to carbon dioxide, and the aortic arch was perfused with phosphate buffer solution (PBS), excised, and processed for histological examination.

### Human tissue sample

Human tissue samples were provided by the tissue bank of the National Center for Tumor Diseases (NCT, Heidelberg, Germany) following the regulations of the tissue bank and the approval of the ethics committee of Heidelberg University on research on humans. Written informed consent was obtained from each patient included in the study. The study protocol conforms to the ethical guidelines of the 1975 Declaration of Helsinki.

Serial 7-µm-thick cryosections were cut from vessel tissue samples embedded in 5% gelatine in a cryostat from 8 subjects with diagnosed atherosclerotic changes (mean age ± SEM, 63 ± 14.8 years) and 3 control subjects (63 ± 5.4 years) without atherosclerotic changes. The tissue specimens originated from amputated limbs; surgical indications are indicated in Supplemental Table S2. Medication with HMG-CoA reductase inhibitors including the daily dose is also indicated in Supplemental Table S2.

### Immunofluorescence analysis

Longitudinal or cross cryosections (10-µm thickness) of the murine aorta or carotid artery were incubated at first with a blocking buffer (5% donkey serum, #017–000-121, Dianova, in PBS-T containing 0.05% Triton-X-100) in a humidified chamber at room temperature for 1 h. The primary endothelial cell marker antibody anti-CD31 (1:50, sc-18916; Santa Cruz Biotechnology, Germany) and the monocyte/macrophage marker antibody anti-F4/80 (1:100, MCA497GA, Bio-Rad Laboratories, Germany) were administered overnight at 4 °C. The corresponding secondary antibodies (1:100, donkey anti-rat AlexaFluor 488 [712-546-150] and donkey anti-rat Dylight 549 [712-585-153]; Dianova, Germany) were subsequently incubated for 1 h at room temperature. In addition, the tissue sections were stained according to standard procedures for MAC387 immunopositive infiltrating macrophages (1:100, ab22506, Abcam, UK) and CD31 (1:100, M0823, Agilent Dako, USA) using donkey-anti-mouse cy3 [715-166-020] and donkey anti-mouse cy2 [715-225-150] as secondary antibodies (1:100, both from Dianova, Germany), respectively. Cell nuclei were stained with DAPI according to standard protocols. Mowiol 4–88 (Merck Millipore) was used as a mounting medium.

Atherosclerotic plaques were identified by haematoxylin and eosin (H&E) staining according to standard protocols and/or using a Movat’s pentachrome staining kit (Dianova, Germany) according to the manufacturer’s instructions. Microscopy and visualization were performed using an Olympus Spinning Disc Confocal microscope and the Olympus Xcellence imaging software (Olympus Europa SE & Co. KG, Hamburg, Germany).

### MALDI MS imaging

For better sectioning, all tissue samples were embedded in a metal mold with 5% gelatine by freezing [24]. Sections with a thickness of 10 µm were mounted on a microscope glass slides (SuperFrost, Thermo Scientific Menzel Gläser) using a cryostat Microm HM550 (Thermo Fisher Scientific, Germany) at a temperature of − 21 °C and stored at − 80 °C until analysis. For analysis, tissues were defrosted in a desiccator, covered with 80 µL of 2,5-dihydroxybenzoic acid (30 mg/mL in acetone:water (1:1) with 0.1% TFA) in one passage with a flow rate of 5 µl/min, a gas pressure of 1 bar, and a tracking speed of 350 U/min using an ultrafine matrix preparation sprayer (SMALDIPrep, TransMIT GmbH, Giessen, Germany). The samples were measured with an atmospheric-pressure MALDI imaging ion source (AP-SMALDI10 and AP-SMALDI5 AF, TransMIT GmbH, Giessen, Germany) coupled to an orbital trapping mass spectrometer (Q Exactive and Q Exactive HF, Thermo Fisher Scientific GmbH, Bremen, Germany) [45]. Measurements were performed in positive-ion mode with 30 laser pulses per pixel using a mass range of *m*/*z* 300–1200 and a mass resolution of *R* = 140,000 or *R* = 240,000 @ *m*/*z* 200. Internal mass calibration was achieved using the lock-mass feature of the orbital trapping mass spectrometer resulting in a mass accuracy of ± 3 ppm. Murine samples were measured with a spatial resolution (i.e., step size) of 7 µm. Depending on the region of interest, human tissue samples were measured with a spatial resolution between 5 and 15 µm per pixel as described in detail in Supplemental Table 2. For lipid characterization on tissue, MS experiments were performed but since signal intensities of the detected markers were too low, the experiments were not successful.

### Data processing and statistical analysis

Based on literature search [16, 28, 51], the lipid classes cholesterol esters (CE), lysophosphatidylcholines (LPC), lysophosphatidylethanolamines (LPE), and cholesterol derivatives (CD) were chosen for this study because these lipid classes play a crucial role in atherosclerosis plaque development and progression. The generated MALDI MSI data, however, contains much more information, e.g., other lipid classes and smaller metabolites not considered in this study.

A list of 551 lipid species was composed and downloaded on September 5, 2017, from the LIPID MAPS database website. Exact *m*/*z* values were calculated using different adducts (H^+^, Na^+^, K^+^, and NH_4_^+^) for these species, and MS images were generated with a bin width of Δ(*m*/*z)* 0.01 using MSiReader V09-1 [44]. MS images showing characteristic patterns of plaque distribution at aortic bifurcations were selected for further analysis. The ApoE^−/−^ mice tissue samples were compared to each other, and only those *m*/*z* values which were found in all samples of > 40-week-old mice and which showed no signal in the WT control samples were selected as potential markers. After calculating mass errors of potential markers using the in-house developed software package Mirion V3 [40], signals with a mass error of less than 3 ppm were selected. Human tissue samples were handled the same way with the difference that due to the lipid variability each measurement was considered individually. A more detailed description of the data analysis is given in the Supplementary Data. In addition, a list of oxidized lipid species [1, 8, 13, 19, 21, 27, 36, 42, 43] was compiled from literature and *m*/*z* values were generated. From this list, only oxCE (18:2) and 9(S)-HODE CE were found in the measurement data set. Oxidized lipids were thus excluded from PCA because we superficially looked at some interesting oxidized lipids and did not perform an in-depth investigation in this regard.

For PCA of the murine tissue samples, intensities of the potential markers were exported and normalized to the total ion current (TIC) of each measurement. Intensities of non-selected signals were set to zero. The PCA was performed using the software package Perseus (version 1.4.1.3, Max Planck Institute of Biochemistry, Martinsried, Germany). The nonparametric Kruskal–Wallis test was used to determine if any of the intensity means significantly differed from each other by calculating *p*-values; *p*-values < 0.05 were considered statistically significant.

All measurements performed on mice tissue samples (ApoE^−/−^ and WT) were carried out using equal measurement conditions (matrix preparation, pixel size, spot size, laser pulses, etc.). Under the given conditions, the MALDI-MSI data of the murine samples can be considered semi-quantitative.

Since the human tissue samples were measured with different spatial distributions, the intensities of the detected lipids were set to 1, for lipids detected with low intensity to 0.5, and for not detected lipids to 0 for the PCA analysis using the software package Perseus. The human tissues samples were divided into two groups (AT and control). Of all detected lipids, only those fulfilling the prerequisite, that 70% of the samples in one group need to have a value greater than 0, were subjected to PCA. The *p*-values were calculated similarly to the mouse data.

## Results

### Murine tissue samples

The ApoE^−/−^ mouse as an established atherosclerosis model was first used to identify possible lipid markers for the subsequent evaluation of human atherosclerotic vessel samples. Atherosclerotic plaques were identified in the aorta on each tissue slide by H&E and/or Movat’s pentachrome staining accompanied by positive immunofluorescence staining for pro-inflammatory macrophages in the vessel wall (exemplified in Supplemental Figure S1). Within identified plaque areas, regions of interest (ROIs) were further subjected to mass spectrometry imaging analysis in the neighbouring sections.

**Fig. 1:**
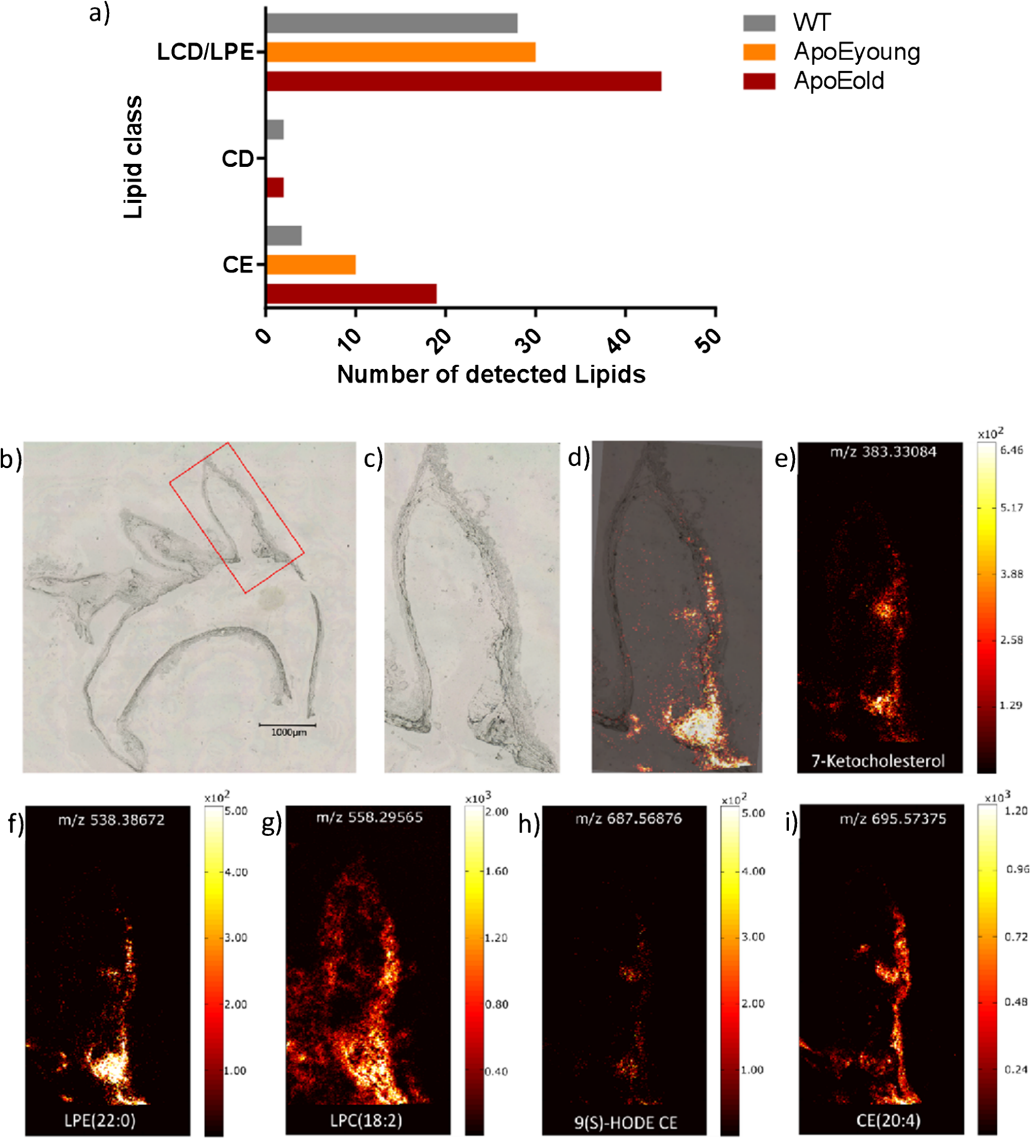
MS imaging of atherosclerotic plaques in the aorta and its branches of ApoE mice. **a** Overall number of detected lipids in the lipid classes of cholesterol ester (CE), cholesterol derivatives (CD), and lysocholestrolderivatives (LCD)/lysophosphatidylethano-lamine (LPE). **b** Exemplary brightfield microscopy overview image of a cross-section of the aortic arch and its branches of an old ApoE knockout mouse (ApoE3, 49 weeks). The red square marks the left subclavian artery, which is shown enlarged in **c**. **d** Overlay of the optical image (**c**) and an MS image representing LPE(22:0) are shown in **f**. **e-i** MS images of the MALDI MSI measurement in positive-ion mode (pixel size 7 µm, 200 × 400 pixels), presenting an example for the overall number of detected lipids shown in **a**

A total number of 65 compounds from the compiled lipid list including adducts were found in the plaque tissue of the older ApoE^−/−^ mice, 31 of which were exclusively found in ApoE^−/−^ but not in the WT mice. Lipid assignments are shown in Supplemental Table S3. In tissue sections of older ApoE^−/−^ mice, the number of detected cholesterol esters was 4.8-fold higher than in those from WT mice and twofold higher than those from young ApoE^−/−^ mice (Fig. 1a). The number of lysophosphatidylcholine (LPC)/lysophosphatidylethanolamine (LPE) lipids, representing most of the detected lipids, was found to be 1.5-fold in tissue sections of older ApoE^−/−^ mice as compared to WT or young ApoE^−/−^ mice (Fig. 1a). For each considered lipid class, examples found to be significantly enriched in the aortic branch and carotid artery of the older ApoE^−/−^ mice are shown in Fig. 1d–i and Supplemental Figure S2c-g, respectively.

**Fig. 2:**
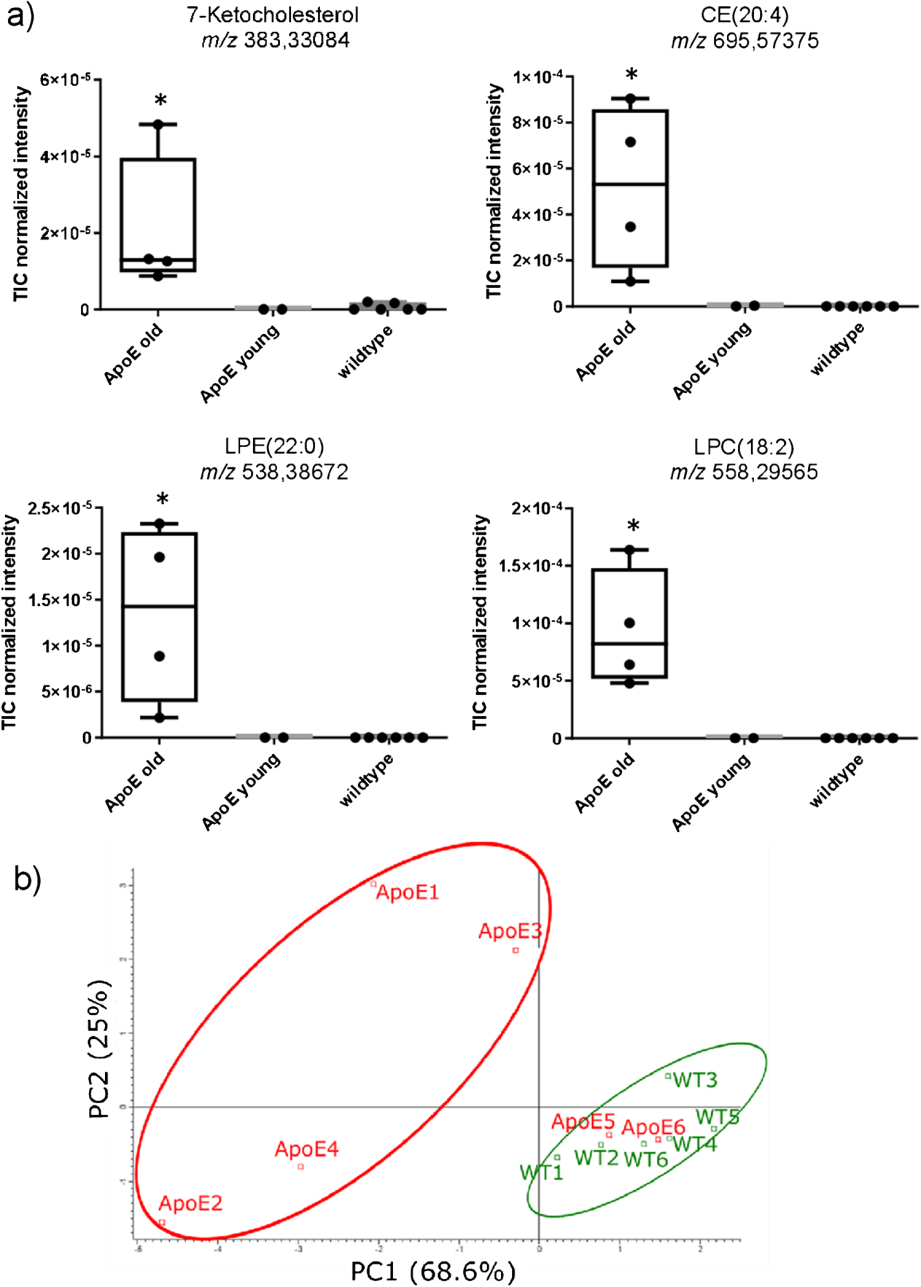
Representative lipids of lipid classes exclusively detected in old ApoE knockout mice and principal component analysis. **a** Statistical summary of the intensities normalized to the total ion current (TIC) for the indicated lipids in the arterial sections of old ApoE^−/−^ (49–60 weeks, *n* = 4), young ApoE^−/−^ (14–15 weeks, *n* = 2), and WT (15–49 weeks, *n* = 6) mice. *p < 0.05 vs. wild-type. **b** Principal component analysis of measured lipids shown in Supplemental Table S3 for WT and ApoE^−/−^ mice. The lipid composition within the plaque areas of the young ApoE^−/−^ mice (ApoE5 and ApoE6) was largely comparable to that of the WT mice

The cholesterol derivative 7-ketocholesterol was detected exclusively in lipid-rich plaque regions in arteries branching off from the aortic arch of old ApoE^−/−^ mice (Fig. 1e, Supplemental Figure S2c). Lysophosphatidylethanolamine LPE(22:0), the degradation product of phosphatidylethanolamine, was detected mainly in the plaque area of the brachiocephalic artery (BCA) (Fig. 1f) and of the right carotid artery (Supplemental Figure S2d). Lysophosphatidylcholine LPC(18:2) was located with the highest intensity in the plaque located at the root of the BCA but also in the whole vessel wall (Fig. 1g) which also held for the right carotid artery (Supplemental Figure S2e). Interestingly, the oxidized lipids 9(S)-HODE CE (exemplarily shown in Fig. 1h and Supplemental Figure S2f) or oxCE (18:2), known to contribute to atherosclerosis progression [52], could be assigned to the *m*/*z* value 687.56876. The cholesterol ester CE(20:4) was not increased in the core plaque area (Fig. 1i). Instead, it accumulated locally in the vessel wall at sites where presumably unidirectional shear stress is low or becomes oscillatory and preferably was located near the branch point and distal to the plaque (Fig. 1i and Supplemental Figure S2g). Statistical summaries of the intensities normalized to the total ion current (TIC) for these lipids in the arterial sections of wild-type, young, and old ApoE^−/−^ mice demonstrated significantly higher intensities in the analysed plaque area of the latter (Fig. 2a).

The (TIC) normalized intensities of the 65 signals detected in arterial specimens of the old ApoE^−/−^ mice were extracted from all MSI measurements done with the same specimens of young ApoE^−/−^ or wild-type mice and subjected to principal component analysis (Fig. 2b). The cluster representing the older ApoE^−/−^ mice (49–56 weeks, ApoE1-4) was found to be separated from that of the young ApoE^−/−^ mice (14–15 weeks, ApoE5,6) and age-matched wild-type mice (48–49 weeks, WT1-6). The lipid composition within the plaque areas of the young ApoE^−/−^ mice was largely comparable to that of the wild-type mice.

### Human tissue samples

**Fig. 3:**
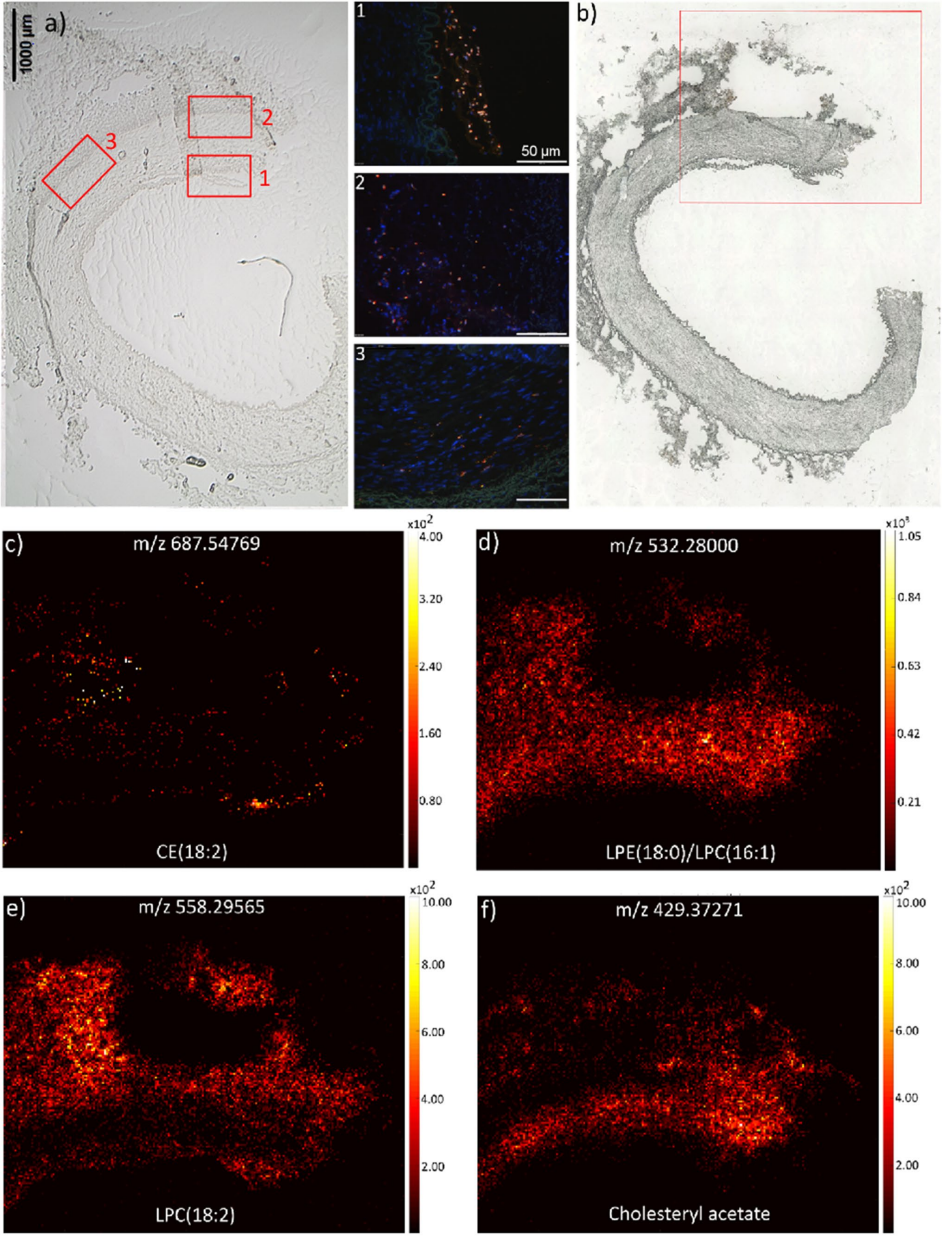
MS imaging of a human artery with atherosclerosis. **a** Exemplary image of a larger peripheral artery resected from the upper thigh (patient AT2), depicting a macrophage infiltrate (red fluorescence) into the atherosclerotic vessel wall (refer to insets 1–3, marked as red squares in the optical image). The blue fluorescence (DAPI) represents the nuclear counterstain. **b** An optical image of the tissue layer is shown and the area (red square), which was measured with MALDI MSI in positive-ion mode. The MS images (**c–f**) show the distribution of the different lipids (pixel size 15 µm, 220 × 185 pixels)

The results of the human tissue measurements show a more diverse lipid distribution. In the direct comparison of the murine and human data, twenty six of the markers, which were exclusively identified in the vessel wall of the older ApoE^−/−^ mice, were also detected in some of the human atherosclerotic plaque samples. Eight of the identified lipid markers, amongst them some cholesterol ester (18:2) and some lysophospholipid species, were also found in the human control samples (please refer to Supplemental Table S4). An exemplary image of a larger peripheral artery resected from the upper thigh (patient AT2, Fig. 3a) and the consecutive serial tissue sections with the marked MS image area is shown in Fig. 3b . Again, the detection of pro-inflammatory macrophages (Fig. 3a, insets 1–3) was used for the identification of the MS imaging region.

Some markers like cholesterol ester (18:2) or lysophosphatidylcholine (18:2), exemplarily shown for patient AT2 in Fig. 3 c and e, that were exclusively found in the arterial specimens of the older ApoE^−/−^ mice, could also be detected in human control samples. Interestingly, high intensities of the ester cholesteryl acetate (Fig. 3f) and the lysolipids LPE(18:0)/LPC(16:1) (Fig. 3d) were detected in macrophage-rich regions of the human atherosclerotic vessel wall (cf. Figure 3A, inset 1).

**Fig. 4:**
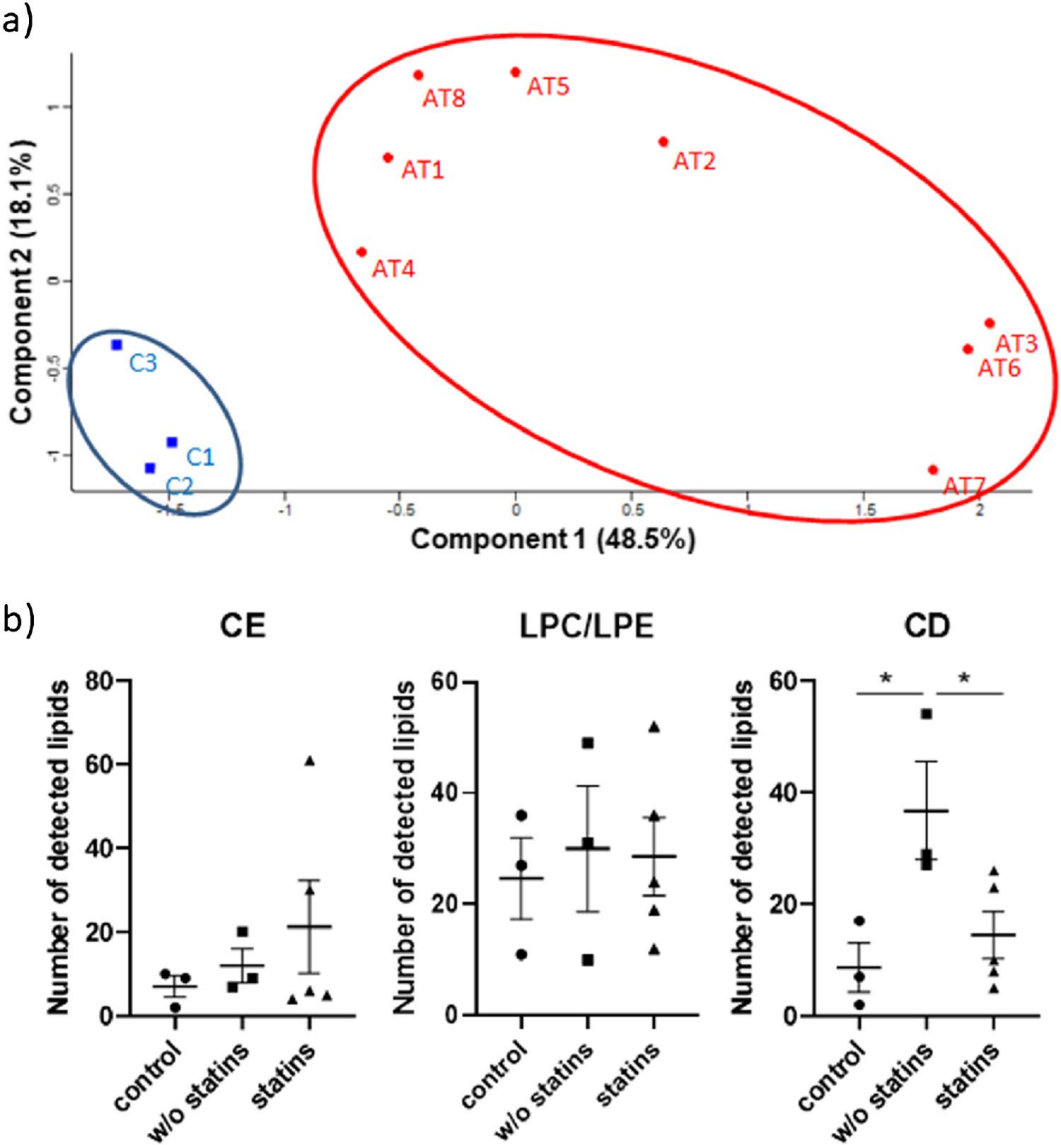
Statistical Analysis of human tissue data. **a** Principal component analysis of 25 m/*z* values found in 70% of the control group (C1–3) and/or the group with atherosclerotic arteries (AT1-8) showing a clear separation between the two groups. **b** Effect of statin therapy on the number of lipids detected in the analysed human tissue samples. Control tissue (*n* = 3), plaque tissue from patients without (*n* = 3), and with HMG-CoA reductase inhibitor treatment (*n* = 5). *p < 0.05 w/o statins vs. control or drug therapy

**Fig. 5:**
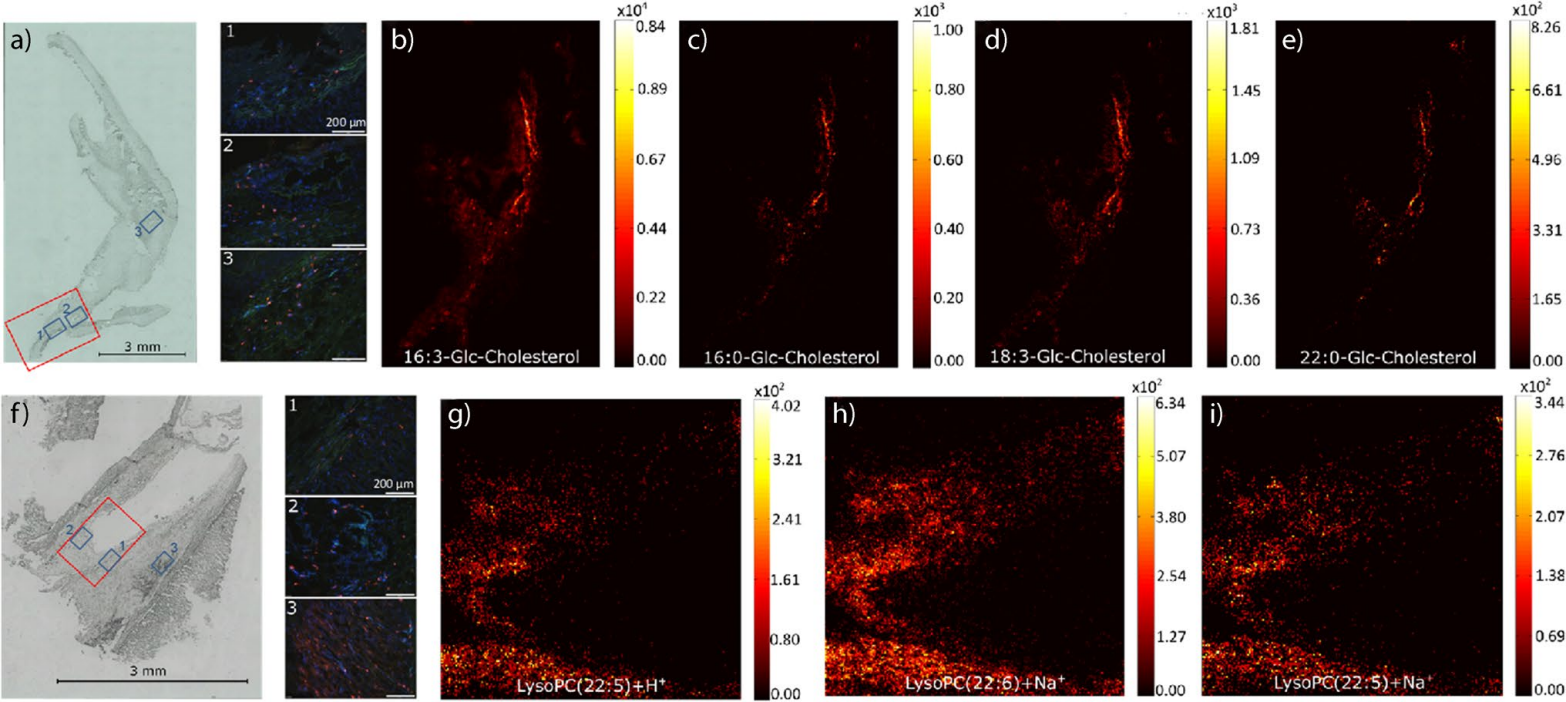
Human atherosclerotic tissue marker. Exemplary presentation of resected lower leg peripheral arteries with arteriosclerotic changes of patient AT6 and AT7. **a** Optical image depicting macrophage infiltrates (red fluorescence) into the atherosclerotic vessel wall (refer to insets 1–3, marked as blue squares in the optical image). The red square shows the area which was measured with MALDI MSI (pixel size 15 µm, 220 × 185 pixels). Exemplary artery MS image of patient AT6 show the marker **b** 16:3-Glc-Cholesterol, **c** 16:0-Glc-Cholesterol, **d** 18:3-Glc-Cholesterol, and **e** 22:0-Glc-Cholesterol (pixel size 7 µm, 220 × 350 pixels). **f** Optical image of the vessel resection of patient AT7 with marked macrophage infiltrates (blue squares 1–3) and the measured MALDI MSI area (red square). Exemplary MS images of the lysophosphatidylcholine marker show **g** LysoPC(22:5) + H^+^, **h** LysoPC(22:6), and **i** LysoPC(22:5) + Na^+^ (pixel size 5 µm, 200 × 200 pixels)

In an extensive data analysis without marker preselection based on mouse data, in total, 317 m/*z* values were found in all human tissue samples and subjected to supervised principal component analysis. Samples were divided into the group with atherosclerotic arteries (AT) and tissue with no significant pathological findings (control). The list of 317 m/*z* values was reduced by the prerequisite that the considered *m*/*z* value must be detected in 70% of the samples of at least one group. PCA of the remaining 25 m/*z* values shows a clear separation between the control and atherosclerotic tissue samples (Fig. 4a). Seven of these *m*/*z* values can be considered statistically significant atherosclerotic tissue markers; one species shows at least a trend towards significance (Supplemental Table S5). Amongst these, the lysophosphatidylcholine species LPC(22:5) and LPC(22:6) have already been identified as markers for the older atherosclerotic ApoE^−/−^ mice (Fig. 5). Four glucosylated cholesterol species, the esterified acyl steryl glucosides 16:0, 16:3, 18:3, and 22:0, not being detected in the murine atherosclerotic tissue, were identified as statistically significant human atherosclerotic markers (Fig. 5, Supplemental Figure S3).

Additionally, a significant difference in the lipid composition of patients receiving lipid-lowering drugs compared to patients without a comparable medication was detected (Fig. 4b). The analysed tissue samples of the five patients receiving an HMG-CoA reductase inhibitor showed significantly lower amounts of cholesterol derivatives (CDs) due to the cholesterol-lowering effect of the drugs as compared to patients receiving no such treatment.

## Discussion

The atherosclerosis-prone ApoE^−/−^ mouse is one of the most widely used murine models for the study of atherosclerosis [15]. The lesion distribution in these mice is similar to humans, with a predominance in the aortic root, carotid artery, and other branches of the aorta [39], so that initiation and progression of plaque formation have been extensively studied in these mice [54]. This monogenetic murine model has also been widely used with high reproducibility to identify metabolic oxidative stress markers in the context of cardiovascular disease [11]. Despite the accelerated atherosclerosis development, a major limitation of the ApoE^−/−^ mouse model is that the lesions rarely rupture and hence do not lead to thrombosis, whereas vascular occlusion is common in humans [39]. In our study, we used MALDI MSI for the direct comparison of murine and human atherosclerotic plaques to determine universally applicable atherosclerotic plaque biomarkers. In addition, a separate data analysis of the human MSI data was performed to determine markers for human atherosclerotic samples.

Using principal component analysis, we found two distinguishable clusters between matching arterial vessel specimens of older ApoE^−/−^ mice and age-matched wild-type littermate control animals. The young ApoE^−/−^ mice showed a lipid composition more comparable to that of the wild-type mice. When getting older, the increase in the severity of hypercholesterolemia and plasma CE levels plays critical roles in the pathogenesis of atherosclerosis in these animals, as it alters the cellular lipid composition like it has been described recently for the development of an impaired skin barrier function in the same mouse model [34]. Very recently, a total of 25 aortic plaque-specific lipid species have been identified by comparing LDLR^−/−^ and ApoE^−/−^ mice fed a high-fat diet for 16 weeks using MALDI MSI. Tissue from wild-type C57BL/6 mice was not analysed but 11 lipid species were consistently detected in atherosclerotic plaques of both mouse strain [6]. Fourteen of these 25 lipids were also identified in our analyses (see Supplemental Table S3). Interestingly, LPE(22:0) specific for atherosclerotic ApoE^−/−^ mice in our study is positively associated with stable coronary artery disease evaluated by plasma lipidomic analysis [35]. Cholesterol ester CE(18:2), additionally identified in our study as ApoE-specific plaque biomarker, was also previously detected in aortic roots of ApoE^−/−^ mice at 60 weeks of age and in femoral arteries of humans with peripheral artery occlusive disease using MSI [56]. The accumulation of cholesterol ester in the arterial intima is one of the main characteristics of macrophage foam cells that reinforce the development of atherosclerotic plaques [16].

9-HODE, the stable oxidation product of linoleic acid in CE, is also known to contribute to the progression of atherosclerosis and the risk of clinical events such as myocardial infarction or stroke [52]. In this late stage of atherosclerosis, the nonenzymatically generated pro-inflammatory 9-HODE increased apoptosis leading to a fragile, acellular plaque [52]. 7-Ketocholestrol, another identified oxysterol in our study, is believed to play an important role in plaque development as it has more potent pro-atherogenic effects than cholesterol [49]. All other lipid species, in addition to the 31 selected in our study, could also be detected in wild-type mice and therefore do not represent specific MALDI MSI detectable lipid biomarkers for atherosclerosis, even though lysophospholipids are known to be involved in the development and progression of the disease [30].

Unfortunately, under the given conditions, a quantitative statistical analysis for lipids in individual layers of the vessel was not possible. So far, only a few studies using MALDI MSI analysis of human arteries have been published (summarized in [41]). The lysophosphatidylcholine species LPC(22:5) and LPC(22:6) were exclusively identified in the older ApoE^−/−^ mice and were also identified as a human atherosclerotic tissue marker. LysoPCs are products of phospholipase A2 (PLA2) enzyme activity [10]. High lysophospholipid content destabilizes membranes. Lipoprotein-associated PLA2 levels have been described to be associated with stroke and atherosclerosis [23]. Some of these presumably specific atherosclerotic markers identified in the older ApoE^−/−^ mice like cholesterol ester (18:2) and lysophospholipid species were also found in human control samples. This illustrates that the human vascular specimens show a more diverse patient-dependent lipid distribution, which is at variance with the vascular specimens derived from the monogenetic ApoE^−/−^ mice. However, it confirms previous findings that clinically relevant atherosclerotic plaques contain a variable mix of lipids [2]. This may be since different stages of atherosclerosis progression lead to larger differences in their lipid profiles. Moreover, diet and lifestyle, i.e., a diet rich in fat and sugar, and smoking [5], as well medication of the patients with cholesterol-lowering drugs [9] influence the lipid profile and hence the results.

However, based on a principal component analysis, we were able to identify four more statistically significant atherosclerotic tissue markers in the human data set which fulfil the prerequisite of being detected in more than 70% of the samples of the atherosclerotic patient group. To the best of our knowledge, these acetylated colesterylsteryl glucosides which belong to the group of sterol lipids are sterol derivatives that have not been described in the context of atherosclerosis before. They belong to the complex group of glycolipids or steryl glycosides and are common plant phytosterol lipids [37] but also have been relatively recently found in various human tissues in significant amounts [4]. Here they serve, e.g., as storage forms of sterols but are also important for the physical stability of the cell membrane [17]. Interestingly, acyl steryl glucosides have been found to stimulate macrophages [48]. Moreover, the supposed acyltransferases involved in the biosynthesis of steryl acyl glucosides require exogenous acyl lipids such as glycerophospholipids as fatty acid sources for acylation [48]. This might explain the observed high content of lysophospholipids destabilizing cellular membranes. On the other hand, it is also possible that glycated lipids accumulated to significant extents in the cells may not be detoxified through the actions of glyoxalase 1 [32]. In our study, colesterylsteryl acyl glucosides could almost be exclusively detected in human atherosclerotic tissue. In this context, it is important to note that higher levels of glycated lipids as advanced glycation end products (AGEs) were associated with a plaque rupture phenotype, typically not observed in mouse models [32].

Additionally, within the human samples, a significantly lower number of cholesterol derivatives, like oxysterols, were detected in vessels of patients receiving HMG-CoA reductase inhibitor treatment. Oxysterols are major constituents of oxLD involved in the pathogenesis of atherosclerotic lesion formation through cholesterol accumulation in macrophage or vascular smooth muscle cell–derived foam cells [29]. HMG-CoA reductase inhibitors also potently inhibit the primary cellular acyl-CoA: cholesterol O-acyltransferase (ACAT) isoform 1, thereby inhibiting esterification of cholesterol and ultimately reducing the accumulation and number of lipid species in the foam cell [3].

The imaged human atherosclerotic vessels in our study originate from amputated limbs. It must also be considered that the human control tissue samples were, of course, free from atherosclerotic changes but originate from amputations due to an injury or disease which of course could influence the immune response leading to differences in the vessel microenvironment and subsequently lipid composition. Therefore, it is not surprising that some of the identified human atherosclerotic tissue markers were also detected in the human control samples.

In summary, our study identified several atherosclerosis-specific lipid biomarkers but demonstrated that a comparison between atherosclerotic vessels originating from a monogenetic mouse model and humans with differences in genetic background, diet, and lifestyle is rather difficult and may lead to wrong conclusions. Signal intensities of less abundant lipids in high spatial resolution MSI experiments are often quite low, making this approach difficult and time-consuming especially since this analysis must be performed on every lipid species. Therefore, the lipid species in this study could only be assigned (to one or more lipids) but not unequivocally identified. We nonetheless show that high-resolution MALDI mass spectrometry imaging is a valid method to comprehensively investigate the lipid profile of atherosclerotic plaques derived from both mice and men.

## Supplementary Information

Below is the link to the electronic supplementary material.

Supplementary file1 (PDF 1285 KB)
