# Supplementary material for: Comparative lipid profiling of murine and human atherosclerotic plaques using high-resolution MALDI MSI
Source: Pflugers Arch. 2021 Nov 19;474(2):231–42. doi: 10.1007/s00424-021-02643-x (PMC8766400; doi:10.1007/s00424-021-02643-x)
Supplement: Supplementary file 1 — Supplementary file1 (PDF 1285 KB) [file 424_2021_2643_MOESM1_ESM.pdf]

Supplemental Information

Materials and Methods

Data processing and statistical analysis

A list of lipids of interest was downloaded using the link < https://lipidmaps.org following the listed steps:

- Navigate to Resource and then Classification
- Chose the lipid species of interest (for example Sterol Lipids [ST] (W) --> Sterols [ST01]--> Cholesterol and derivatives [ST0101])
- At the end of the page, you can download the results as CSV

For each murine tissue measurement, 551 lipids (downloaded as described) were considered and m/z values were calculated with H+, Na+, K+, and NH4+ adducts. In total 2204 MS images were generated and manually screened for each measurement. All m/z images showing ion intensity exclusively on the tissue with a distribution comparable to the optical image were selected for further analysis. After mass error calculation and statistical analysis, only those lipids listed in Table S3 remain. For each considered lipid class one example was chosen for Figures 1 and 2. The same procedure was applied to the human tissue sample with the difference that every measurement was considered individually. That means every lipid which was found with a mass error of less than 3 ppm was considered as a possible marker (even if those lipids were found in the control samples) while in the murine tissue samples only those lipids which were found in all old ApoE mice and additionally not found in the control samples were considered as markers.

After data analysis, we followed two approaches.

1. We compared murine and human datasets (presented in Table S4) to see whether the results are comparable and if there are lipids that can be considered as a marker in murine and human atherosclerotic tissue samples

2. We performed an individual statistical analysis based on the human results to see whether a trend or specific marker can be detected (shown in Figure 5)

Table S1: Murine vessel tissue samples of male ApoE$^{-/-}$ (apolipoprotein E) knockout and WT (wildtype) mice.

**Mouse ID number**	**Localization**	**Age (weeks)**
ApoE1	aortic arch	56
ApoE2	1st carotid	60
ApoE3	aortic arch	49
ApoE4	aortic arch	56
ApoE5	aortic arch	15
ApoE6	aortic arch	14
WT1	left subclavian	48
WT2	left subclavian	48
WT3	left subclavian	48
WT4	aortic arch	15
WT5	aortic arch	49
WT6	aortic arch	15

Table S2: Human samples (AT1-8, artery with atherosclerotic changes; C1-3, no significant pathological findings).

**Patient ID number**	**Localisation**	**Gender**	**Age**	**Medication**	**Surgical indication / vascular finding**	Pixel size [µm]	Mass Resolution @ *m/z* 200
AT1	lower leg	female	64	Atorvastatin (40mg)	pAVK, wound healing disorder / muscular artery with lipoidotic plaques and arteriosclerotic changes	5	140000
AT2	upper thigh	male	38	x	car accident / muscular artery with low lipoidotic plaques and arteriosclerotic changes	15	140000
AT3	upper thigh	female	85	Atorvastatin (40 mg)	pAVK / peripheral artery with arteriosclerotic changes, no calcification	7	140000
AT4	lower leg	male	78	Pravastatin (20mg)	pAVK, necrosis / peripheral artery with arteriosclerotic changes (media hypertrophy)	5	140000
AT5	upper thigh	female	71	Atorvastatin (20mg)	pAVK / peripheral artery with arteriosclerotic changes and focal metaplastic ossification	7	140000
AT6	lower leg	male	53	x	chronic osteomyelitis / resected vessel wall, peripheral larger artery with arteriosclerotic changes	7	140000
AT7	lower leg	male	56	Atorvastatin (20mg)	chronic osteomyelitis, pAVK / peripheral artery with arteriosclerotic changes (intimal fibrosis and medial hypertrophy)	5	140000
AT8	lower leg	male	66	x	Critical ischemia / peripheral artery with arteriosclerotic changes (medial hypertrophy)	7	240000
C1	lower leg	female	55	x	Hip endoprosthesis infection / peripheral artery with no significant pathological findings	5	140000
C3	lower leg	female	73	x	demarcation, muscle necrosis / peripheral artery with no significant pathological findings	7	240000

C2	lower leg	female	60	x	decompensated ischemia of the foot in recurrent occlusions of the popliteal artery and lower leg arteries / peripheral artery with no significant pathological findings	7	240000

Table S3: List of detected lipids in ApoE$^{-/-}$ and WT mice (d= detected, nd= not detected, dwli= detected with low intensity). Sub-grouping in columns is based on localisation for ApoE$^{-/-}$ (1st carotid, brachiocephalic artery, aortic arch) and WT (brachiocephalic artery, aortic arch). Some lipids have also been identified and described as aortic plaque specific by Cao et al. (Cao, J, Goossens, P, Martin-Lorenzo, M, et al., Atheroma-Specific Lipids in ldlr–/– and apoe–/– Mice Using 2D and 3D Matrix-Assisted Laser Desorption/Ionization Mass Spectrometry Imaging, Journal of the American Society for Mass Spectrometry, 2020;Epub 2020, July 27). Mass error > 3 ppm to the theoretical mass. Markers exclusively found in older ApoE$^{-/-}$ mice are summarized in Table S4.

			**ApoE**			**WT**		
**m/z**	**Adduct**	**Lipid**	**4/1/2**	**3**	**5/6**	**1/2/3**	**4/5/6**	**Comment**
628.60271	NH_4^+	15:0 Cholesteryl ester	d	d	d	d	d	uneven C chain
645.55810	Na^+	16:1 Cholesteryl ester	d	d	nd	nd	nd	
647.57375	Na^+	16:0 Cholesteryl ester	d	d	d/nd	nd	nd	
663.54769	K^+	16:0 Cholesteryl ester	d	d	nd	nd	nd	
666.61836	NH_4^+	18:2 Cholesteryl ester	d	d	nd	nd	nd	
		16:1 Stigmasteryl ester						
		16:2 Sitosteryl ester						
669.55810	Na^+	18:3 Cholesteryl ester	d	d	d/nd	nd	nd	
		16:2 Stigmasteryl ester						
		16:3 Sitosteryl ester						
671.57375	Na^+	18:2 Cholesteryl ester	d	d	d/nd	nd	nd	
		zymosteryl oleate						
		16:1 Stigmasteryl ester						
		16:2 Sitosteryl ester						
673.58940	Na^+	18:1 Cholesteryl ester	d	d	d/nd	nd	nd	
		16:0 Stigmasteryl ester						
		16:1 Sitosteryl ester						
675.60505	Na^+	18:0 Cholesteryl ester<br>16:0 Sitosteryl ester	d	d	nd	nd	nd	
687.54769	K^+	18:2 Cholesteryl ester	d	d	d/nd	nd	nd	
		16:1 Stigmasteryl ester						

		16:2 Sitosteryl ester						
687.56867	Na^+	OxCE (18:2)<br>9(S)-HODE cholesteryl ester	d	d	nd	nd	nd	
693.55810	Na^+	20:5 Cholesteryl ester	d	d	nd	nd	nd	
695.57375	Na^+	20:4 Cholesteryl ester<br>18:3 Stigmasteryl ester	d	d	d/nd	nd	nd	
697.58940	Na^+	20:3 Cholesteryl ester<br>18:2 Stigmasteryl ester	d	d	nd	nd	nd	
697.59181	H^+	22:6 Cholesteryl ester	d	d	nd	nd	nd	
701.62070	Na^+	20:1 Cholesteryl ester<br>18:0 Stigmasteryl ester<br>18:1 Sitosteryl ester	d	d	nd	nd	nd	
705.58164	H^+	Cholesteryl 11-hydroperoxy-eicosatetraenoate	d	d	d	d	d	
723.60505	Na^+	22:4 Cholesteryl ester<br>20:3 Stigmasteryl ester	d	d	nd	nd	nd	
727.56358	Na^+	Cholesteryl 11-hydroperoxy-eicosatetraenoate	d	d	d	d	d	
743.53752	K^+	Cholesteryl 11-hydroperoxy-eicosatetraenoate	d	d	d	nd	d	in ApoE1 mass error > 3 ppm
454.29282	H^+	LysoPE(16:0)	dwli	d	nd	nd	nd	
468.30847	H^+	LysoPC(14:0)	dwli	d	nd	nd	nd	
480.30847	H^+	LysoPE(18:1)	d	d	d/nd	d	nd	
482.32412	H^+	LysoPE(18:0)	d	d	d/nd	d	d	
490.29041	Na^+	LysoPC(14:0)	d	d	nd	nd	nd	
494.32412	Na^+	LysoPE(18:0)<br>LysoPC(16:1)	d	d	d/nd	d	d	

496.33977	H^+	LysoPC(16:0)	d	d	d	d	d	Also identified by Cao et al.
502.29282	H^+	LysoPE(20:4)	d	d	d/nd	d	d	
504.30606	Na^+	LysoPE(18:0)	d	d	d/nd	dwli	dwli	
510.35542	H^+	LysoPE(20:0)	d	d	nd	dwli	dwli	Also identified by Cao et al.
516.30606	Na^+	LysoPC(16:1)	d	d	d	d	d	
520.33977	H^+	LysoPC(18:2)	d	d	d	d	d	Also identified by Cao et al.
522.35542	H^+	LysoPC(18:1)	d	d	d/nd	d	d	Also identified by Cao et al.
524.37107	H^+	LysoPC(18:0)	d	d	d/nd	d	d	Also identified by Cao et al.
532.33736	Na^+	LysoPE(20:0)	d	d	d/nd	dwli	dwli	
538.38672	H^+	LysoPE(22:0)	d	d	nd	nd	nd	Also identified by Cao et al.
540.30606	Na^+	LysoPC(18:3)	d	d	nd	nd	nd	
542.32171	Na^+	LysoPC(18:2)	d	d	d/nd	d	d	Also identified by Cao et al.
544.33736	Na^+	LysoPC(18:1)	d	d	d/nd	d	d	Also identified by Cao et al.
546.35301	Na^+	LysoPC(18:0)	d	d	d	d	d	Also identified by Cao et al.
558.29565	K^+	LysoPC(18:2)	d	d	nd	nd	nd	
560.31130	K^+	LysoPC(18:1)	d	d	d/nd	d	d	Also identified by Cao et al.
562.32695	K^+	LysoPC(18:0)	d	d	d/nd	d	d	Also identified by Cao et al.
566.32171	Na^+	LysoPC(20:4)	d	d	d/nd	d	d	
568.33736	Na^+	LysoPC(20:3)	d	d	d/nd	nd	nd	
568.33977	H^+	LysoPC(22:6)	d	d	d/nd	dwli	dwli	
570.35301	Na^+	LysoPC(20:2)	d	d	d/nd	nd	nd	

570.35542	H^+	LysoPC(22:5)	d	d	d/nd	nd	nd	
572.36866	Na^+	LysoPC(20:1)	d	d	nd	nd	nd	
574.38431	Na^+	LysoPC(20:0)	d	d	nd	nd	nd	
590.32171	Na^+	LysoPC(22:6)	d	d	d/nd	dwli	dwli	
592.33736	Na^+	LysoPC(22:5)	d	dwli	dwli/nd	nd	nd	
518.32171	Na^+	LysoPC(16:0)	d	d	d	d	d	Also identified by Cao et al.
524.27476	Na^+	LysoPE(20:4)	d	d	d	d	d	
526.29282	H^+	LysoPE(22:6)	d	d	d/nd	d	d	
528.30847	H^+	LysoPE(22:5)	d	d	d/nd	d	d	
530.32412	H^+	LysoPE(22:4)	d	d	nd	d	dwli	
532.28000	K^+	LysoPE(18:0)	d	d	nd	nd	nd	
		LysoPC(16:1)						
548.27476	Na^+	LysoPE(22:6)	d	d	d	d	d	
548.31130	K^+	LysoPE(20:0)	d	d	nd	nd	nd	
550.29041	Na^+	LysoPE(22:5)	d	d	d	d	d	
552.30606	Na^+	LysoPE(22:4)	d	d	dwli/nd	dwli	nd	
560.36866	Na^+	LysoPE(22:0)	d	d	nd	nd	nd	
581.42892	NH_4^+	LysoPE(24:1)	d	d	nd	nd	nd	
383.33084	H^+	3,5-cholestadien-7-one	d	d	nd	dwli	nd	
		4,6-cholestadienone						
		7-dehydro-desmosterol						
		Zymosterone						
		1,4-cholestadienone						
	H^+- H_2O	7-oxo-cholesterol						
385.34649	H^+	Cholestenone	d	d	nd	dwli	dwli	
		Desmosterol						
		Zymosterol						
		7-dehydrocholesterol						

		22-dehydrocholesterol						
		5alpha-cholesta-7,24-dien-3beta-ol						
		8-Dehydrocholesterol						
		Lumisterol 3						
		3-ketocholesterol						
		Cystosterol						
		22Z-dehydrocholesterol						
		1-(5alpha)-cholestenone						
		5, 20(22)-cholestadienol						
		4,6-cholestadienol						

Table S4: Markers exclusively detected in ApoE$^{-/-}$ mice compared to human atherosclerotic tissue and human control samples. Sub-grouping in columns is based on localisation for ApoE$^{-/-}$ mice. Dark blue: detected in human atherosclerotic samples, Light blue: detected in human control sample, Red: mass error > 3ppm to the theoretical mass (WT, wild-type control mice).

			ApoE			WT		Human atherosclerotic tissue								Human control tissue		
**m/z**	**Adduct**	**Lipid**	**4/1/2**	**3**	**5/6**	**1/2/3**	**4/5/6**	**AT2**	**AT3**	**AT6**	**AT7**	**AT1**	**AT5**	**AT4**	**AT8**	**Con1**	**Con2**	**Con3**
645.55810	Na^+	16:1 Cholesteryl ester	d	d	nd	nd	nd	nd	nd	nd	d	nd	nd	nd	dwli	nd	nd	nd
647.57375	Na^+	16:0 Cholesteryl ester	d	d	d/nd	nd	nd	nd	nd	nd	d	nd	nd	nd	d	nd	nd	nd
663.54769	K^+	16:0 Cholesteryl ester	d	d	nd	nd	nd	nd	nd	nd	d	nd	nd	nd	d	nd	nd	nd
666.61836	NH_4^+	18:2 Cholesteryl ester<br>16:1 Stigmasteryl ester<br>16:2 Sitosteryl ester	d	d	nd	nd	nd	nd	nd	nd	d	nd	nd	nd	d	nd	nd	nd
669.55810	Na^+	18:3 Cholesteryl ester<br>16:2 Stigmasteryl ester<br>16:3 Sitosteryl ester	d	d	d/nd	nd	nd	nd	nd	nd	d	nd	nd	nd	d	nd	nd	nd
671.57375	Na^+	18:2 Cholesteryl ester<br>zymosteryl oleate<br>16:1 Stigmasteryl ester<br>16:2 Sitosteryl ester	d	d	d/nd	nd	nd	d	d	nd	d	nd	d	d	nd	d	nd	nd
673.58940	Na^+	16:0 Stigmasteryl ester<br>16:1 Sitosteryl ester	d	d	d/nd	nd	nd	nd	nd	nd	d	nd	nd	d	nd	d	nd	nd
687.54769	K^+	18:2 Cholesteryl ester	d	d	d/nd	nd	nd	d	nd	nd	d	nd	nd	d	d	d	d	nd

		16:1 Stigmasteryl ester<br>16:2 Sitosteryl ester																
693.55810	Na^+	20:5 Cholesteryl ester	d	d	nd	nd	nd	nd	nd	nd	d	nd	nd	d	nd	nd	nd	nd
695.57375	Na^+	20:4 Cholesteryl ester<br>18:3 Stigmasteryl ester	d	d	d/nd	nd	nd	nd	nd	nd	d	nd	nd	d	nd	nd	nd	nd
697.58940	Na^+	20:3 Cholesteryl ester<br>18:2 Stigmasteryl ester	d	d	nd	nd	nd	nd	nd	nd	d	nd	nd	d	nd	nd	nd	nd
697.59181	H^+	22:6 Cholesteryl ester	d	d	nd	nd	nd	nd	nd	nd	d	nd	nd	d	nd	nd	nd	nd
701.62070	Na^+	20:1 Cholesteryl ester<br>18:0 Stigmasteryl ester<br>18:1 Sitosteryl ester	d	d	nd	nd	nd	nd	nd	nd	d	nd	nd	nd	nd	nd	nd	nd
723.60505	Na^+	22:4 Cholesteryl ester<br>20:3 Stigmasteryl ester	d	d	nd	nd	nd	nd	nd	nd	d	nd	nd	d	nd	nd	nd	nd
468.30847	H^+	LysoPC(14:0)	dwli	d	nd	nd	nd	d	nd	nd	nd	nd	d	d	d	dwli	dwli	nd
490.29041	Na^+	LysoPC(14:0)	d	d	nd	nd	nd	d	nd	nd	nd	nd	d	d	nd	nd	nd	nd
558.29565	K^+	LysoPC(18:2)	d	d	nd	nd	nd	d	d	d	d	d	d	d	d	d	d	d
568.33736	Na^+	LysoPC(20:3)	d	d	d/nd	nd	nd	d	dwli	nd	d	nd	d	d	d	d	dwli	nd
570.35301	Na^+	LysoPC(20:2)	d	d	d/nd	nd	nd	d	dwli	nd	d	nd	d	d	d	dwli	nd	nd
570.35542	H^+	LysoPC(22:5)	d	d	d/nd	nd	nd	d	dwli	nd	d	nd	d	d	d	dwli	nd	nd
572.36866	Na^+	LysoPC(20:1)	d	d	nd	nd	nd	d	nd	nd	d	nd	d	dwli	nd	dwli	nd	nd
592.33736	Na^+	LysoPC(22:5)	d	dwli	dwli/nd	nd	nd	d	dwli	dwli	d	nd	d	d	nd	nd	nd	nd
532.28000	K^+	LysoPE(18:0)<br>LysoPC(16:1)	d	d	nd	nd	nd	d	nd	nd	nd	d	d	d	d	dwli	nd	nd

548.31130	K^+	LysoPE(20:0)	d	d	nd	nd	nd	nd	nd	nd	nd	d	nd	d	nd	nd	nd	nd
581.42892	NH_4^+	LysoPE(24:1)	d	d	nd	nd	nd	nd	nd	nd	d	nd	nd	nd	nd	nd	nd	nd
383.33084	H^+	3,5-cholestadien-7-one	d	d	nd	dwli	nd	nd	nd	nd	d	nd	nd	d	nd	nd	nd	nd
		4,6-cholestadienone																
		7-dehydro-desmosterol																
		Zymosterone																
		1,4-cholestadienone																
	H^+-H_2O	7-oxo-cholesterol																

Table S5: List of human atherosclerotic lipid markers with fulfilling the prerequisite of being detected in at least 70% of the human atherosclerotic tissue samples and not detected in more than one control sample (d= detected, nd= not detected, dwli= detected with low intensity). Mass error > 3 ppm to the theoretical mass. Two human AT tissue markers have also been identified in the old ApoE–/– mice. P-values less than 0.05 were regarded as statistically significant and calculated using the two-tailed t-test with the following values for nd=0, dwli=0.5, and d=1 (AT1-8, artery with atherosclerotic changes; C1-3, no significant pathological findings).

			Human atherosclerotic tissue								Control tissue				
**m/z**	**Adduct**	**Lipid**	**AT1**	**AT2**	**AT3**	**AT4**	**AT5**	**AT6**	**AT7**	**AT8**	**C1**	**C2**	**C3**	**Comment**	**P-value**
570.35542	H^+	LysoPC(22:5)	nd	d	dwli	d	d	nd	d	d	dwli	nd	nd	old ApoE marker	0.1046
590.32171	Na^+	LysoPC(22:6)	dwli	d	d	d	d	dwli	d	d	nd	nd	nd		< 0.05
592.33736	Na^+	LysoPC(22:5)	nd	d	dwli	d	d	dwli	d	nd	nd	nd	nd	old ApoE marker	< 0.05
803.57963	Na^+	16:3-Glc-Cholesterol	d	d	d	d	d	d	d	d	nd	nd	nd		< 0.05
825.60051	K^+	16:0-Glc-Cholesterol	d	d	d	nd	d	d	d	d	nd	nd	dwli		< 0.05
831.61093	Na^+	18:3-Glc-Cholesterol	d	d	d	nd	d	d	nd	d	nd	nd	nd		< 0.05
909.69441	K^+	22:0-Glc-Cholesterol	d	d	d	d	d	d	nd	d	nd	nd	dwli		< 0.05

Figure S1

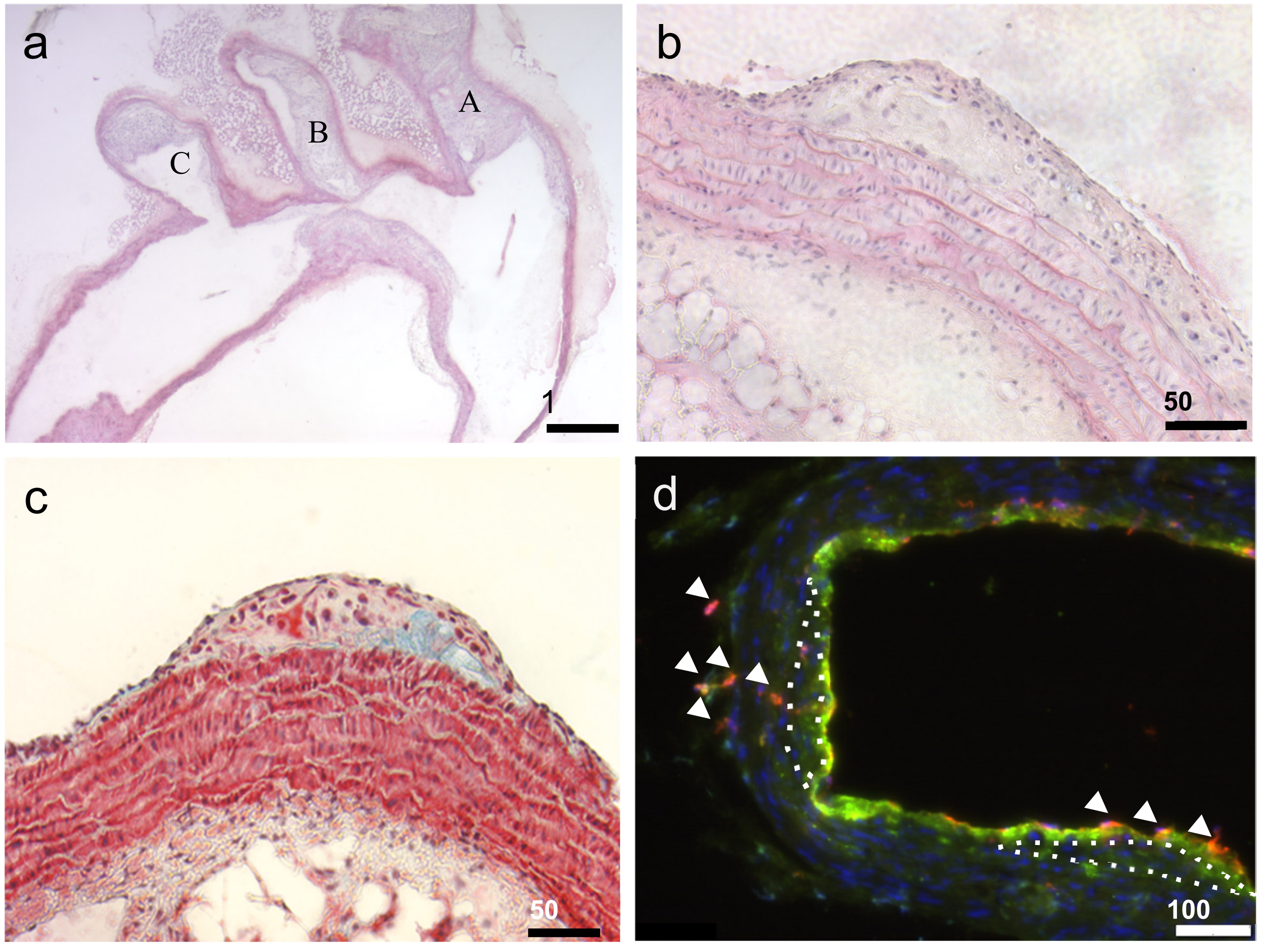

Detection of atherosclerotic plaques in ApoE–/– mice. (a) Representative bright-field image of a longitudinal section from the aortic arch of a male ApoE–/– mouse (56 weeks) stained with H&E (A, brachiocephalic artery; B, left common carotid; C, left subclavian artery). (b) Representative bright-field image of an atherosclerotic plaque stained with H&E and (c) Movat Pentachrome for histological demonstration of collagen (yellow), elastin and nuclei (blue to black), muscle (red), mucin (bright blue), and fibrin (bright red). (d) Exemplary picture, depicting macrophage infiltrates (red fluorescence, arrows) in the atherosclerotic plaque area (dashed line) Endothelial cell surface marker CD31 (green fluorescence), DAPI nuclear counterstain (blue fluorescence). Scale bars as indicated.

Figure S2

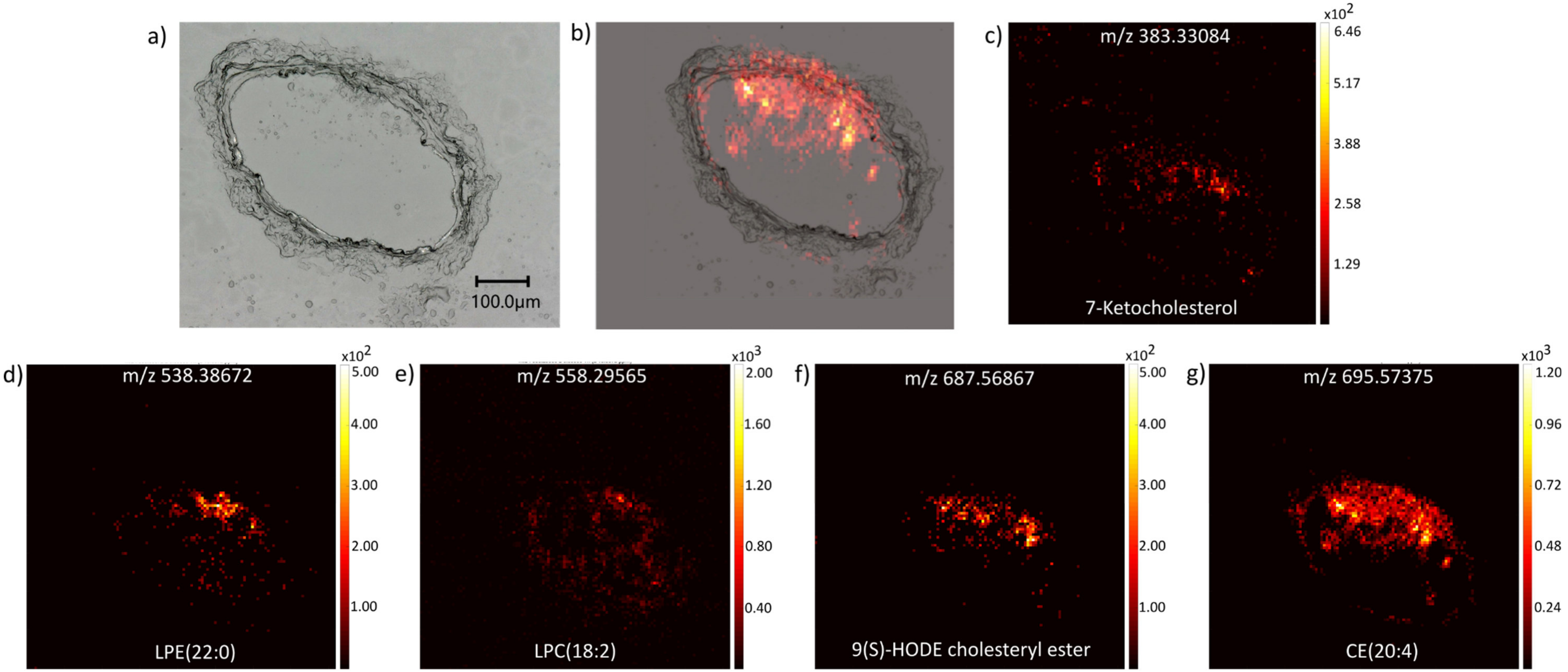

MALDI MSI measurement in the positive-ion mode of the right carotid artery of ApoE mice (ApoE2, 60 weeks). (a) Optical image of the carotid artery. (b) Exemplary overlay of the optical and the CE(20:4) MS image. The MS images (c-f) show the distribution of the different types of lipids (pixel size 7 µm, 117x115 pixels).

Figure S3

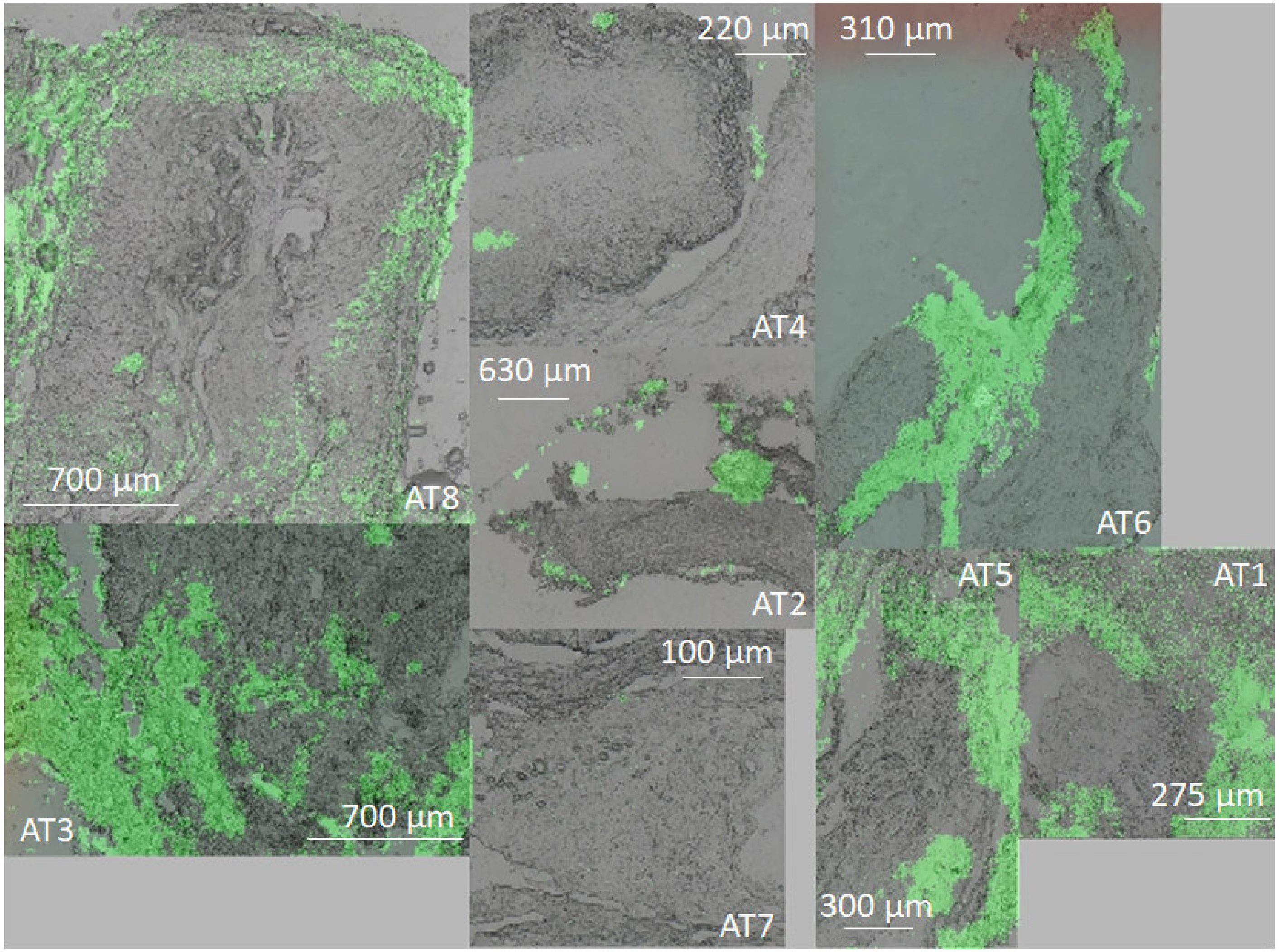

MS images of the identified human atherosclerotic tissue marker 18:3-Glc-Cholesterol (m/z 803.57963). Overlay with the optic microscopic image of the area measured in all human vessel resections (patient AT1-8) with a spatial resolution between 5 and 15 µm per pixel.
